# Delayed diagnosis in children with autism spectrum disorder or intellectual disability

**DOI:** 10.1590/1980-5764-DN-2024-0279

**Published:** 2025-09-01

**Authors:** Janaína Aparecida de Oliveira Augusto, Carolina Kulcsar Caravieri, Rodrigo Genaro Arduini, Décio Brunoni, Sylvia Maria Ciasca, Maria Cristina Triguero Veloz Teixeira

**Affiliations:** 1 Universidade Presbiteriana Mackenzie, São Paulo SP, Brazil.; 2 Universidade Estadual de Campinas, Campinas SP, Brazil.

**Keywords:** Intellectual Disability, Autism Spectrum Disorder, Delayed Diagnosis, Health Knowledge, Attitudes, Practice, Parents, Deficiência Intelectual, Transtorno do Espectro Autista, Diagnóstico Tardio, Conhecimentos, Atitudes e Prática em Saúde, Pais

## Abstract

In the developing countries, there is a long delay between parents’ first concerns and the diagnosis of neurodevelopmental disorders such as autism spectrum disorder (ASD) and intellectual disability (ID).

**Objective:**

To verify the time interval between the first concerns reported by parents and the diagnosis of ASD and ID in a pediatric neurology outpatient clinic in the public health network in Brazil, and to verify any association between indicators of cognitive functioning and behavioral and emotional problems.

**Methods:**

One hundred and six children diagnosed with ID (n=69) and ASD (n=37), with a mean age of 10.03 years; standard deviation (SD)=2.2. The inclusion criteria were diagnoses of ID and ASD, and the exclusion criteria were comorbid conditions such as attention-deficit/hyperactivity disorder and specific learning disorder.

**Results:**

The mean age of reported first symptoms was 29.9 months (SD=19.5) for ASD and 37.9 months (SD=19.5) for ID; and the mean time from first concerns to diagnosis of ID was 6.8 years (SD=2.2) and 6.6 years (SD=2.7) for ASD. Children with ID scored significantly lower than those with ASD cognitive functioning (t [97]=-16.22, p<0.001). Children with ID had higher mean scores for externalizing problems (M=62.20; SD=11) and those with ASD had higher mean scores for internalizing problems (M=66.71; SD=10.01).

**Conclusion:**

The results indicated a late diagnosis in the sample and, given the mental health care received, the data highlight the need for training primary care professionals to identify and diagnose ASD and ID at an early stage.

## RESUMO

Nos países em desenvolvimento há um longo intervalo de tempo entre as primeiras preocupações relatadas pelos pais e o diagnóstico de transtornos do neurodesenvolvimento como transtorno do espectro autista (TEA) e deficiência intelectual (DI).

**Objetivo:**

Verificar o intervalo de tempo entre as primeiras preocupações relatadas pelos pais e o diagnóstico de TEA e DI em um serviço público brasileiro de neurologia pediátrica e verificar se há associação entre indicadores de funcionamento cognitivo e problemas comportamentais e emocionais.

**Métodos:**

Cento e seis crianças com diagnóstico de DI (n=69) e TEA (n=37), com idade média de 10,03 anos; desvio padrão (DP)=2,2. Os critérios de inclusão foram o diagnóstico de DI e TEA, foram excluídas comorbidades com o transtorno de déficit de atenção/hiperatividade e transtorno específico de aprendizagem.

**Resultados:**

A idade média relatada dos primeiros sintomas foi de 29,9 meses (DP=19,5) para TEA e 37,9 meses (DP=19,5) para DI; o tempo médio entre as primeiras preocupações e o diagnóstico de TEA foi de 6,6 anos (DP=2,7) e do DI foi de 6,8 anos (DP=2,2). Crianças com DI tiveram médias mais altas para problemas de comportamento externalizante (M=62,20; DP=11) e as com TEA tiveram médias mais altas para problemas de comportamento internalizantes (M=66,71; DP=10,01).

**Conclusão:**

Os resultados indicaram um diagnóstico tardio na amostra e, considerando-se as intervenções em saúde mental recebidas, os dados revelam a necessidade de capacitação dos profissionais da atenção básica para identificar e diagnosticar precocemente TEA e DI.

## INTRODUCTION

There is scientific evidence about the benefits of early diagnosis and intervention in neurodevelopmental disorders such as autism spectrum disorder (ASD) and intellectual disability (ID)^1,2,3^. The literature shows that low- and middle-income countries still have high rates of late diagnosis of these disorders^4,5^. However, a recent review study found no statistically significant differences between low-/middle-income and high-income countries in terms of the average first concern reported by parents and the average age of diagnosis of ASD^6^.

Despite the results found in this review, previous studies have shown that parents’ first concerns about suspected ID or ASD may be reported before 24 months of age^1,2,3,7^. In countries such as the USA, the average age at diagnosis of ASD is 50 to 56 months, and in the UK it is 55 months^2,8,9^. In Brazil, there is little data on the average age of diagnosis of ASD or ID; a study conducted in the Southern Region, which followed the development of 4,231 babies during the first 24 months of life, found that, of the 195 children identified with developmental delay, 151 were diagnosed with ID between the ages of seven and eight^4^. Regarding ASD, a Brazilian study found that the time elapsed between the identification of the first parental concerns and the diagnosis of ASD was approximately five years^10^.

Various factors such as comorbidities, mild clinical presentation, behavioral problems, difficulties in accessing health care services, and others can contribute to late diagnosis^1,2,3^. A survey in the US found that children whose initial concerns are related to language delay tend to be diagnosed with ASD earlier, children with behavioral concerns have an average age of diagnosis of 87.9 months, while those whose primary concerns are learning disabilities have an average age of diagnosis for ASD of 69.7 months^6,11^. The presence of ID is a factor that influences the average time from first manifestation to final diagnosis; in general, parents of children with cognitive impairment tend to report first concerns earlier than those of children without such impairments^11^.

High-income countries have adopted the use of screening tools to identify risk signs for ASD and ID, which can contribute to early diagnosis during pediatric visits^12,13^. However, the use of these screening tools is still limited in Brazil. The challenges of implementing screening tools include the high cost of training, limited access to the tools, and lack of adequately qualified professionals, etc.^14^.

Late diagnosis of ASD or ID may contribute to the child missing the opportunity to be included in early interventions that can help reduce symptoms and behavioral and emotional problems, in addition to resulting in worse cognitive and adaptive functioning compared to those who receive treatment^1,2,3,6^.

Understanding the length of time between parental concern and diagnosis of ASD or ID, and its impact on the child’s behavior and adaptive functioning, is essential for the development of public policies aimed at raising awareness and enhancing the training of healthcare professionals. Such initiatives may promote earlier diagnoses of both disorders. Moreover, compared to ASD, there are few studies that examine the time between parents’ first concerns regarding suspicion of ID and the age of the child’s diagnosis. This study seeks to address the existing gap in the literature.

The objectives of this study were to examine the time interval between the first concerns reported by parents and the diagnosis of ASD or ID in a pediatric neurology outpatient clinic in a public hospital in Brazil. In addition, the study assessed whether there was an association between age at diagnosis and indicators of cognitive and adaptive functioning and behavioral problems.

## METHODS

The study design was exploratory and a retrospective study with a non-probability sample of children evaluated at the Laboratory for Research in Disturbances and Learning and Attention Disorders (DISAPRE) between 2017 and 2019, and their parents. During this period, 348 children and adolescents aged six to 16 years were evaluated. The main complaints reported by the parents were: developmental delays, learning difficulties and behavioral problems. Those referred to the service came from schools, health professionals (public and private) and other health services. The children were evaluated by a multidisciplinary team composed of a psychologist, psychopedagogue, speech therapist and neuropediatrician.

We adopted two inclusion criteria: a) diagnosis of ASD or ID provided by the DISAPRE mental health team, b) age between six and 14 years, c) complete assessment in the database using the Vineland Adaptive Behavior Scales (Third Edition) and the Autistic Traits Assessment. The diagnoses of ASD and ID were established by a multidisciplinary team based on the criteria of the Diagnostic and Statistical Manual of Mental Disorders, 5^th^ Edition (DSM-5)^15^ and a clinical, neuropsychological and socioemotional evaluation of the multidisciplinary team (the tests used in this evaluation are described in the instruments section). The age at first concern was determined based on parental report.

Children with comorbidities or diagnoses of other neurodevelopmental disorders, such as attention-deficit/hyperactivity disorder and specific learning disorders, were excluded from the study. After applying the inclusion and exclusion criteria, 106 children and adolescents (30.4%) were eligible for participation in the study. The study was approved by the Ethics Committee of Mackenzie Presbyterian University (CAAE: 4.632.789).

### Instruments

- The Wechsler Intelligence Scale for Children (WISC-IV)^16^: assesses intelligence; the sum of the four indices (verbal comprehension, perceptual reasoning, working memory, and processing speed) that make up the scale yields the intelligence quotient (IQ);
- Child Behavior Checklist for ages 6–18 (CBCL/6-18)^17^: assesses behavioral and emotional problems based on parental report. Items are grouped into empirically based syndrome scales (e.g., anxious/depressed, withdrawn/depressed, somatic complaints, attention problems, and rule-breaking behavior) and DSM-oriented scales (e.g., depressive problems, anxiety problems, attention deficit and oppositional defiant disorders), internalizing problems, externalizing problems, and total problems;
- Vineland Adaptive Behavior Scale^18^: assesses adaptive functioning based on parent report. It is divided into four domains: communication, autonomy, socialization, and motor skills;
- Autistic Traits Assessment (ATA)^19^: assesses signs of ASD based on parent report. It consists of 23 subscales that assess deficits in social interaction, manipulation of the environment, use of people around them, resistance to change, search for rigid order, lack of eye contact, inexpressive mime, sleep disturbances, change in diet, difficulty controlling sphincters, exploration of objects, inappropriate use of objects, lack of attention, lack of interest in learning, lack of initiative, changes in language and communication, lack of skills and knowledge, inappropriate reactions to frustration, hyperactivity/hypoactivity, stereotyped and repetitive movements, ignores danger, and onset of symptoms before 36 months;
- Anamnesis to verify the child’s age, the age when parents and caregivers first reported concerns regarding suspected signs of the disorders (e.g., language delay, neuropsychomotor delay, and behavior problems), as well as the interventions and assessments conducted prior to diagnosis.

### Data analysis

The data was analyzed using JASP Statistics, version 0.15. Descriptive analyses were performed to characterize the sample in terms of gender, age, age at first parental concerns, and age at diagnosis. The Mann-Whitney test was used to test for differences between diagnoses (ASD and ID) in the time interval between first parental concerns and mean age at diagnosis. The t-test was also employed to assess differences in cognitive functioning and behavioral and emotional problems between the groups, and to verify differences in adaptive functioning between the groups. For this test, the effect size was assessed using Cohen’s d-test (0.20-small magnitude, 0.50-medium magnitude, and 0.80-large magnitude). Results with p≤0.05 were considered statistically significant.

## RESULTS

The sample of the study consisted of 106 children diagnosed with ID (n=69) and ASD (n=37), with a mean age of 10.04 years (SD=2.18) and 35.8% male in the ID group (Table 1). The mean IQ of the ID group was 61.87 (SD=5.2) while the mean IQ of the ASD group was 87.95 (SD=17.0). Of the total sample, 92.5% were enrolled in public schools, with 73.6% between 2^nd^ and 5^th^ grade. Additionally, 26.8% of the parents of children with ID reported having completed a high school education (Table 1).

**Table 1:** Sociodemographic characteristics by participants (N=106).

Children		Intellectual disability N=69	Autism Spectrum Disorder N=37	U (p-value)
**Sex N (%)**	Female	31 (29.2)	1 (0.9)	
	Male	38 (35.8)	36 (34.1)	
**Age – mean (SD)**		10.4 (2.18)	9.35 (2.23)	
**IQ – mean (SD)**		61.87 (5.2)	87.95 (17.0)	
**Type of school**	Public	66 (62.2)	32 (30.3)	
	Private	3 (2.8)	5 (4.7)	
**Educational level N (%)**	1^st^ grade	2 (1.9)	3 (2.8)	
	2^nd^ grade	9 (8.5)	11 (10.4)	
	3^rd^ grade	18 (17.0)	3 (2.8)	
	4^th^ grade	13 (12.3)	8 (7.5)	
	5^th^ grade	10 (9.4)	6 (5.7)	
	6^th^ grade	6 (5.7)	2 (1.9)	
	7^th^ grade	6 (5.7)	2 (1.9)	
	8^th^ grade	1 (0.9)	2 (1.9)	
	9^th^ grade	4 (3.8)	-	
**First parental concerns – mean in months (SD)**		37.95 (19.5)	29.93 (19.5)	434.50 (0.76)
**Age at diagnosis – mean in years (SD)**		6.8 (2.23)	6.6 (2.70)	358.50 (0.62)
**Parents educational level N (%)**	Elementary school	13 (15.9)	9 (11.0)	
	Middle school	13 (15.9)	9 (11.0)	
	High school	22 (26.8)	9 (11.0)	
	Higher education	4 (4.9)	3 (3.7)	

Abbreviations: SD, standard deviation; IQ, intelligence quotient; *U*, Mann-Whitney test; p, significance level.

Regarding the first concerns reported by caregivers, the mean age at the time of first concern for ASD children was 29.93 months (standard deviation—SD=19.5) and, for those with ID, 37.95 months (SD=19.5). No statistically significant differences were identified between the groups (U=434.50, p=0.76). However, the mean time between parents’ first concern and diagnosis was 6.8 years (SD=2.23) for ID group and 6.6 years (SD=2.7) for ASD group. No statistically significant differences in mean time were found between the groups (U=358.50, p=0.62 (Table 1).

The anamnesis revealed important data regarding interventions prior to diagnosis. For the ID group, the most common were speech therapy (31.8%), psychotherapy (27.4%), and neurological medical services (22.6%). For the ASD group, the most common interventions were psychiatric services (14.2%), speech therapy (12.3%), and psychotherapy (11.3%). In addition, 17% of the children with ID and 12.3% of those with ASD were using medication at the time of the specialized evaluation at DISAPRE (Table 2). Furthermore, 16% of the children with ID were referred to the service by the school, 14.1% by neurologists and 4.7% by pediatricians. In the ASD group, 17.9% were referred by a neurologist, 8.5% by the school, and 1.9% by a pediatrician (Table 2).

**Table 2:** Description of the professionals who referred the children to the health service.

Professionals	Group	n (%)
Neurologists	ID	15 (14.1)
	ASD	19 (17.9)
School staff	ID	17 (16)
	ASD	9 (8.5)
Psychologist	ID	11 (10.4)
	ASD	2 (1.9)
Speech therapist	ID	8 (7.5)
	ASD	-
Psychiatrist	ID	3 (2.8)
	ASD	2 (1.9)
Pediatrician	ID	5 (4.7)
	ASD	2 (1.9)
Occupational therapist	ID	-
	ASD	1 (0.9)
Geneticist	ID	7 (6.6)
	ASD	1 (0.9)

Abbreviations: ID, intellectual disability; ASD, autism spectrum disorder.

Children with ID had lower mean scores on all intellectual assessment indices compared to the ASD group, and a large effect size was observed (Table 3). No significant correlations were found between IQ and mean age at diagnosis in either the ID group (r=0.193; p=0.253) or the ASD group (r=0.011; p=0.960).

**Table 3:** Comparison of scores in the intellectual assessment test according to the groups.

Measure	Group (N)	Average	SD	*t*(p-value)	Cohen’s d
IQ	ID (69)	61.87	5.24	-16.22 (<0.001)	-3.55
	ASD (37)	87.90	17.00		
VCI	ID (69)	68.20	6.87	-10.10 (<0.001)	-2.21
	ASD (37)	93,57	18,19		
POI	ID (69)	69.03	8.98	-12.88 (<0.001)	-2.82
	ASD (37)	98.87	13.65		
WMI	ID (69)	63.54	9.94	-10.31 (<0.001)	-2.25
	ASD (37)	87.40	11.96		
PSI	ID (69)	66.97	10.55	-9.99 (<0.001)	-2.19
	ASD (37)	89.73	10.03		

Abbreviations: ID, intellectual disability; ASD, autism spectrum disorder; SD, standard deviation; IQ, intelligence quotient; VCI, verbal comprehension index; POI, perceptual organization index; WMO, working memory index; PSI, processing speed index; *t*, t-test.

Notes: p significance level (p < 0.001); Cohen’s d: effect size.

No statistically significant differences were found in adaptive functioning between children with ASD and those with ID, but the ID group had lower mean scores in communication (mean=40.17; SD=12.9) and socialization (mean=47.01; SD=12.30). In Vineland, lower scores indicate a greater deficit in adaptive functioning (Table 4). A weak statistically significant correlation was found between mean age at diagnosis and adaptive social skills in the ID group (r=0.375; p=0.022). No significant correlations were found in other domains in the ID group (communication r=-0.153; p=0.366 / autonomy r=-0.056; p=0.741) or in the ASD group (communication r=-0.718; p=0.069 / autonomy r=-0.121; p=0.819 / social r=0.491; p=0.323).

**Table 4:** Comparison of scores in the adaptive functioning assessment test according to the groups.

Vineland	Group (N)	Average	SD	*t*(p-value)	Cohen’s *d*
Communication	ID (69)	40.17	12.90	-1.79 (0.077)	-0.40
	ASD (13)	47.85	19.86		
Autonomy	ID (69)	36.88	15.30	-0.684 (0.496)	0.15
	ASD (13)	33.58	15.80		
Socialization	ID (69)	47.01	12.30	-1.55 (0.125)	-0.35
	ASD (13)	52.83	9.66		
Total score	ID (69)	38.11	10.70	-0.345 (0.732)	-0.08
	ASD (13)	39.27	7.80		

Abbreviations: ID, intellectual disability; ASD, autism spectrum disorder; SD, standard deviation; *t*, t-test.

Notes: p significance level; Cohen’s d: effect size.

No statistically significant differences were found between the two groups (ID and ASD) for emotional and behavioral problems on the CBCL. However, the ASD group showed higher mean scores for internalizing problems (mean=66.71; SD=10.01; t [83]=-1.44, p=0.15), and no statistically significant correlations were found between internalizing problems and mean age at diagnosis in the ID group (r=0.199; p=0.300) and ASD (r=0.083; p=0.720). The ID group had higher mean scores for externalizing problems (mean=62.20; SD=11, t [83]=1.55, p=0.13). No significant correlations were found between externalizing problems and mean age at diagnosis in either the ID group (r=-0.001; p=0.996) or the ASD groups (r=0.074; p=0.749).

## DISCUSSION

The purpose of the present study was to evaluate the time interval between the first parent-reported concerns and the final diagnosis of ASD or ID, and to examine associations between the mean age of diagnosis cognitive and adaptive functioning and behavioral problems.

Unlike what has been observed in developed countries, where the average age of first family concerns occurs before 24 months^1,2,3,7,14^. In the present study, the results indicated that the first concerns occurred later (between 29.9 and 37.9 months). Previous studies have described signs such as lack of eye contact, neuropsychomotor and language delays are the first concerns reported by parents^3,6,7^.

In addition, the presence of late diagnosis (approximately 84 months) was observed in this sample, confirming the results of other Brazilian studies on these disorders^4,14,20,21^. A better outcome was reported in a recent review, which found an average of 52.60 months in high-income countries and 60.38 months in middle/low-income countries. However, these authors caution about the delay in the interval between reporting of first concerns and final diagnosis of ASD in the studies reviewed^6^.

The variability in the age of ASD diagnosis has been linked to factors such as mild signs, cognitive level, language delays, etc.^22,23^. Late diagnosis may lead to inappropriate interventions, which, in turn may contribute to severe symptoms and/or delay in identifying comorbidities, in addition to worsening behavioral and emotional problems^2,3,6^.

Another aspect that stands out in our study is that, although pediatricians are the professionals responsible for following up and monitoring the development of children, in this study we observed a low rate of referral by these professionals to the service. The Brazilian Ministry of Health provides guidelines for the diagnosis of ASD^24^ and ID^25^, but our results most likely reflect the Brazilian context, where there are still health professionals without adequate training for the early detection of signs of ASD and ID.

These results underscore the importance of investing in the training of primary care professionals to enhance their ability to recognize early signs of neurodevelopmental disorders. The implementation of efficient screening tools, such as the M-CHAT, is essential for early diagnosis. Early detection facilitates timely intervention, which can significantly improve developmental outcomes and mitigate the long-term socioeconomic burdens associated with delayed treatment. Integrating routine screening protocols into primary care visits ensures the early identification of children at risk, facilitating the timely initiation of individualized therapies and support services^12,13,26^.

Additionally, continuous education and training programs should be prioritized to ensure that healthcare providers remain up to date with the latest diagnostic criteria and intervention strategies. Such initiatives can enhance their confidence and competence in conducting developmental screenings and making appropriate referrals. Integrating multidisciplinary teams, including psychologists, speech therapists, and occupational therapists, into primary care settings can foster a more comprehensive approach to early diagnosis and intervention.

Although the results suggest that the children had received previous assessments and interventions, the use of mental health services in this sample does not appear to have contributed to a reduction in the time between the first concern and diagnosis, confirming the need for greater information sharing and dialogue between professionals involved in the assessment^27^.

Previous studies have shown that the use of screening tools to identify developmental delays promotes early diagnosis^12,13,26^. In addition, the pediatrician’s monitoring of the child’s development can help identify, for example, language delays and behavioral and emotional problems, which can serve to identify risk signs for ASD and ID. Initial management by the primary care provider favors referral to speech-language pathology, occupational therapy, or psychology interventions, which are fundamental to both disorders^13^. In ID, studies have suggested the use of behavioral indicators in clinical assessment to map impairments in areas of adaptive functioning, which can inform clinical judgment in making diagnostic decisions. Assessment based on behavioral and adaptive problem indicators could be a possible strategy, especially in the absence of access to appropriate instruments or the unavailability of specialized professionals^28^.

Language delay is the most commonly reported concern by parents of children who are later diagnosed with ASD^6^. Previous studies have reported that parents are often instructed to initiate interventions in areas such as speech therapy or psychology prior to diagnosis, which may benefit the child’s development and aid in the clinical decision to diagnose^8,26^. However, the children in this study were followed by these professionals for years, and this factor did not contribute to an earlier diagnosis.

The study did not find statistically significant differences in behavior problems between children with ASD and children with ID. However, behavioral and emotional problems in children are important predictors of increased parental stress, exacerbating symptoms, and comorbidities, and impact the effectiveness of interventions^9,29^. Findings from other studies suggest that children diagnosed late with ASD have more behavioral and emotional problems than those diagnosed early^8^. In addition, individuals with ID tend to have more externalizing and internalizing problems than typical children^29^.

Notably, most participants were enrolled in public schools, which may be indicative of lower socioeconomic status. Evidence suggests that individuals from economically disadvantaged backgrounds tend to have more difficulty accessing health care, potentially contributing to the delayed diagnoses, as observed in this study^4^.

It is possible that the mild intellectual impairment in the ID group may have contributed to the delay in diagnosis. These results confirm previous studies which reported that mild symptoms contribute to delayed identification, usually at school age^1,7^. On the other hand, in the ASD group, the pattern of cognitive functioning was within the average range. Children with average intelligence and mild signs may not be identified until school age. This may be the case, even though differences in social communication skills and more restricted patterns of behavior may become more apparent due to differences with peers and increased demands in terms of socialization, communication, and adaptive functioning at school^27^.

Analysis of adaptive functioning revealed deficits in both groups, but children with ID had lower scores in communication and socialization. Assessment of adaptive functioning is essential for the diagnosis of ID^15,28^, The delay in diagnosis found in our study may exacerbate the social behavior problems observed in ID, such as difficulty with social judgment, understanding social rules, difficulty making decisions, etc.^15^. On the other hand, individuals with ASD may also have impairments in adaptive functioning. There is evidence that children with ASD and average IQ, as observed in our sample, may have deficits in adaptive functioning, particularly in social skills^30^. The study identified important findings, but several limitations that should be considered when interpreting the results. First, the results cannot be generalized to the general population, since the sample was non-probabilistic and evaluated in a specialized service. Additionally, there was variation in cognitive and behavioral functioning between groups, a lack of data on socioeconomic status and the duration and frequency of previous interventions performed by the participants.

In conclusion, the results of this study indicated the presence of late diagnosis in the sample, which is contrary to national and international guidelines recommending that both diagnoses be made in the first years of the child’s life. The study highlights the urgent need to identify the factors that delay the early diagnosis of severe neurodevelopmental disorders in Brazil.

These findings underscore the critical responsibility of mental health professionals to conduct clinical evaluations when neurodevelopmental disorders such as ASD and ID are suspected. Early and accurate diagnosis is essential for the implementation of evidence-based interventions, which can optimize developmental outcomes, improve long-term prognosis, and enhance quality of life for both children and their families.

## Data Availability

The datasets generated and/or analyzed during the current study are available from the corresponding author upon reasonable request.
